# Supplementary material for: Has a change in established care pathways during the first wave of the COVID-19 pandemic led to an excess death rate in the fragility fracture population? A longitudinal cohort study of 1846 patients
Source: BMJ Open. 2022 May 6;12(5):e058526. doi: 10.1136/bmjopen-2021-058526 (PMC9082727; doi:10.1136/bmjopen-2021-058526)
Supplement: Author's manuscript [file bmjopen-2021-058526.draft_revisions.pdf]

BMJ Open

BMJ Open is committed to open peer review. As part of this commitment we make the peer review history of every article we publish publicly available.

When an article is published we post the peer reviewers' comments and the authors' responses online. We also post the versions of the paper that were used during peer review. These are the versions that the peer review comments apply to.

The versions of the paper that follow are the versions that were submitted during the peer review process. They are not the versions of record or the final published versions. They should not be cited or distributed as the published version of this manuscript.

BMJ Open is an open access journal and the full, final, typeset and author-corrected version of record of the manuscript is available on our site with no access controls, subscription charges or pay-per-view fees (http://bmjopen.bmj.com).

If you have any questions on BMJ Open's open peer review process please email info.bmjopen@bmj.com

BMJ Open

Has a change in established care pathways during the 1st wave of the Covid-19 pandemic led to an excess death rate in the fragility fracture population? A longitudinal cohort study of 1846 patients.

Journal:	*BMJ Open*
Manuscript ID	bmjopen-2021-058526
Article Type:	Original research
Date Submitted by the Author:	09-Nov-2021
Complete List of Authors:	Norrish, Alan; University of Nottingham School of Medicine, Trauma and Orthopaedics; Queen Elizabeth Hospital King's Lynn NHS Foundation Trust<br>Ikram, Adeel; University of Nottingham School of Medicine, Trauma & Orthopaedics; Nottingham University Hospitals NHS Trust<br>Ollivere, Luke; Nottingham University Hospitals NHS Trust<br>Nightingale, Jessica; University of Nottingham School of Medicine, Trauma and Orthopaedics; Nottingham University Hospitals NHS Trust<br>Valdes, A; University of Nottingham School of Medicine; NIHR Nottingham Biomedical Research Centre<br>Ollivere, Benjamin; University of Nottingham School of Medicine, Trauma & Orthopaedics; Nottingham University Hospitals NHS Trust
Keywords:	COVID-19, ORTHOPAEDIC & TRAUMA SURGERY, Hip < ORTHOPAEDIC & TRAUMA SURGERY

SCHOLARONE™
Manuscripts

I, the Submitting Author has the right to grant and does grant on behalf of all authors of the Work (as defined in the below author licence), an exclusive licence and/or a non-exclusive licence for contributions from authors who are: i) UK Crown employees; ii) where BMJ has agreed a CC-BY licence shall apply, and/or iii) in accordance with the terms applicable for US Federal Government officers or employees acting as part of their official duties; on a worldwide, perpetual, irrevocable, royalty-free basis to BMJ Publishing Group Ltd ("BMJ") its licensees and where the relevant Journal is co-owned by BMJ to the co-owners of the Journal, to publish the Work in this journal and any other BMJ products and to exploit all rights, as set out in our licence.

The Submitting Author accepts and understands that any supply made under these terms is made by BMJ to the Submitting Author unless you are acting as an employee on behalf of your employer or a postgraduate student of an affiliated institution which is paying any applicable article publishing charge ("APC") for Open Access articles. Where the Submitting Author wishes to make the Work available on an Open Access basis (and intends to pay the relevant APC), the terms of reuse of such Open Access shall be governed by a Creative Commons licence – details of these licences and which Creative Commons licence will apply to this Work are set out in our licence referred to above.

Other than as permitted in any relevant BMJ Author's Self Archiving Policies, I confirm this Work has not been accepted for publication elsewhere, is not being considered for publication elsewhere and does not duplicate material already published. I confirm all authors consent to publication of this Work and authorise the granting of this licence.

For peer review only

Has a change in established care pathways during the 1st wave of the Covid-19 pandemic led to an excess death rate in the fragility fracture population? A longitudinal cohort study of 1846 patients.

Alan R Norrish [1,2,4], Adeel Ikram[1,2], Luke Ollivere[2], Jessica Nightingale[1,2], Ana M Valdes[1,3], Benjamin J Ollivere[1,2,3]

Affiliations

[1] School of Medicine, University of Nottingham

[2] Nottingham University Hospitals NHS Trust

[3] NIHR Nottingham Biomedical Research Unit (BRC), UK

[4] Queen Elizabeth Hospital, King's Lynn, UK

Corresponding Author:

Mr Adeel Ikram

Specialty Registrar Trauma & Orthopaedics

University of Nottingham Queen's Medical Centre,

Academic Orthopaedics, C Floor, West Block

Nottingham University Hospitals NHS Trust, Derby Road,

Nottingham, NG7 2UH

Email: adeel.ikram@nottingham.ac.uk

Word Count (Excluding title page and references): 2925

Tables: 3

Figures: 2

Key Words: COVID-19, excess death, fragility fracture, hip fracture

Declarations

Ethical Approval – This is an observational study. The study was registered and approved with our local hospital audit department (no. 20-333-C) ethical approval for this study was not needed.

Consent to participate and consent to publish – Consent to participate was not required as this was a retrospective study and collected data was anonymised. Consent to publish was not required, all collected data was anonymised.

Authors Contributions

Alan Norrish ORCID - 0000-0003-3735-1042

Methodology, data analysis and interpretation, original draft preparation review and editing.

Adeel Ikram ORCID - 0000-0002-7520-6949

Methodology, data analysis and interpretation, original draft preparation review and editing.

For peer review only - http://bmjopen.bmj.com/site/about/guidelines.xhtml

Luke Ollivere

Hospital coding database manager, data extraction and acquisition, resources.

Jessica Nightingale ORCID – 0000-0001-8359-5705

Database manager, data extraction and acquisition, resources, reviewed manuscript.

Ana Valdes ORCID- 0000-0003-1141-4471

Conceptualised study, methodology, secured funding, interpretation of data, reviewed manuscript, approved version to be published. Supervised study

Benjamin Ollivere ORCID - 0000-0002-1410-1756

Conceptualised study, methodology, interpretation of data, reviewed manuscript, approved version to be published. Supervised study.

Funding – The research leading to these results received funding from UKRI/MRC Rapid Response COVID trial under grant number (MR/V027883/1).

Conflicts of interest/ Competing interests

- The authors have no relevant financial or non-financial interests to disclose.
- The authors have no conflicts of interest to declare that are relevant to the content of this article.
- All authors certify that they have no affiliations with or involvement in any organisation or entity with any financial interest or non-financial interest in the subject matter or materials discussed in this manuscript.
- The authors have no financial or proprietary interests in any material discussed in this article.

Availability of data and materials

The datasets generated during and/or analysed during the current study are not publicly available due to them containing patient identifiable data within the NHS. But anonymised data are available from the corresponding author on reasonable request.

For peer review only

Has a change in established care pathways during the 1st wave of the Covid-19 pandemic led to an excess death rate in the fragility fracture population? A longitudinal cohort study of 1846 patients.

Abstract

Objective

During the 1st wave of the COVID-19 pandemic, changes to established care pathways and discharge thresholds for patients with fragility fractures were made. This was to increase hospital bed capacity and minimise the inpatient risk of contracting COVID-19. This study aims to identify the excess death rate in this population during the first wave of the pandemic.

Design

A prospective longitudinal cohort study of patients with fragility fractures identified by specific ICD-10 codes. The first wave of the pandemic was defined as the 3-month period between 1st March to 1st June 2020. The control group presenting between 1st March to 1st June 2019.

Setting

Two acute National Health Service (NHS) Hospitals within the East Midlands region of England.

Participants

1846 patients with fragility fractures over the aforementioned two specified matched time points.

Primary and Secondary outcome measures

Four-month mortality of all patients with fragility fractures with a sub analysis of patients with fragility hip fractures.

Results

832 patients with fragility fracture were admitted during the pandemic period (104 diagnosed with COVID-19). 1014 patients presenting with fragility fractures in the control group. Mortality in fragility fracture patients without COVID-19 were significantly higher amongst pandemic period admissions (14.7%) than the pre-pandemic cohort (10.2%), (Hazards-Ratio (HR)=1.86; 95%CI 1.41-2.45; p<0.0001) adjusted for age and sex. Length of stay was shorter during the pandemic period (effect size =-4.2 days 95%CI -5.8, -3.1, p<0.0001). Sub-analysis of fragility hip fracture patients revealed a mortality of 8.4% in the pre-pandemic cohort, and 15.48% during pandemic admissions with no COVID-19 diagnosis, HR=2.08 95%CI 1.11-3.90 p=0.021.

Conclusions

There is a significant increase in excess death, not explained by confirmed COVID-19 infections. Altered care-pathways and aggressive discharge criteria during the pandemic are likely responsible for the increase in excess deaths.

For peer review only - http://bmjopen.bmj.com/site/about/guidelines.xhtml

Article Summary

Strengths and limitations of this study

- This study is one of the largest cohort studies in fragility hip fracture patients investigating the excess mortality in Covid-19 negative patients during the 1st wave of the Covid-19 pandemic.
- Specific ICD-10 codes allowed us to identify the same injury patterns over both groups and a comparative time matched cohort was used as a control.
- The effect of the provision of personal protective equipment and transmission between healthcare staff and patients was not known and this data was not available.
- The redeployment of support staff particularly physiotherapists and orthogeriatric staff for fragility fracture patients may have had an influence on negative outcomes, however specific redeployment data was not available for this study.

Funding

The research leading to these results received funding from UKRI/MRC Rapid Response COVID trial under grant number (MR/V027883/1).

Conflicts of interest/ Competing interests

- The authors have no relevant financial or non-financial interests to disclose.
- The authors have no conflicts of interest to declare that are relevant to the content of this article.
- All authors certify that they have no affiliations with or involvement in any organisation or entity with any financial interest or non-financial interest in the subject matter or materials discussed in this manuscript.
- The authors have no financial or proprietary interests in any material discussed in this article.

Availability of data and materials

The datasets generated and/or analysed during the current study are not publicly available due to them containing patient identifiable data within the NHS. But anonymised data are available from the corresponding author upon reasonable request.

For peer review only

Introduction

The Office of National Statistics in the United Kingdom, states between the 1st March and 30th June 2020 there were 218,837 deaths in England and Wales, of which 50,335 (23%) involved the coronavirus (COVID-19) [1]. At the start of March 2020, the number of deaths per day was below the five-year average, possibly due to mild winter levels of circulating influenza. By the end of the first week of April 2020 this figure was more than double the five-year average, demonstrating the significant impact of COVID-19 on all-cause mortality [1].

The first wave of the COVID-19 pandemic transformed the delivery of orthopaedic services. There was cessation of elective operative practice, a reduction in trauma operative capacity as intensive care bed capacity needed to expand into theatres, and redeployment of all grades of orthopaedic staff (surgeons, medics and allied healthcare professionals) to frontline emergency and intensive care. Established evidenced-based care pathways were modified to reduce length of stay where possible and to lower discharge thresholds to increase bed capacity within hospitals. Following the imposition of a national lockdown, the government advice to the public was to work from home and undertake essential travel only. Following this there was a reduction in the activity of the general population and therefore a reduction in the presentation of major and poly-trauma activity. However, the number of patients presenting with fragility hip fractures did not cease. The World Health Organisation stated in April 2020 that elderly patients are at highest risk from Covid-19 [2], and a recent study demonstrated an increased risk of mortality of 7.8% of people aged over 80 with an age related gradient[3].

Studies have shown an increase in excess deaths by analysis of all-cause mortality, not just due COVID-19 (COVID) infection, during the first wave of the global COVID pandemic [4]. Non-COVID excess deaths are associated with increasing age, with the largest increases in non-COVID deaths being attributed to dementia and frailty. It is well known patients who present with fragility fractures are at risk of poorer outcomes [5,6][1, 2] and outcomes are care pathway dependent.

This study was designed to quantify the excess mortality related to the pandemic in this group following the modification of established care pathways in the light of the first wave. By maintaining long-established evidence-based care pathways for fragility fractures, even in the face of a future wave of the COVID-19 pandemic or future pandemics, excess death in this vulnerable population may be reduced.

Materials and Methods

A retrospective cohort study design identified study and control groups from the same institution, a United Kingdom (UK) hospital group with a major trauma centre and 1700 inpatient beds. Inclusion criteria for both groups were all patients, of all ages, admitted with an ICD-10 code [7] of S72 (fracture of femur), M80 (osteoporosis with current pathological fracture), M96 (intraoperative and postprocedural complications and disorders of musculoskeletal system, not elsewhere classified), W06 (fall from bed), W19 (unspecified fall) and Y79 (orthopaedic devices associated with adverse incidents). These ICD-10 codes were selected to capture patients who were likely presenting with fragility fractures, and were identified from data entered for the

For peer review only - http://bmjopen.bmj.com/site/about/guidelines.xhtml

national Hospital Episode Statistics submission. Two time periods were selected to define groups: (i) the study group were admitted during the 1st wave of the UK COVID-19 pandemic, between 1 March 2020 to 1 June 2020, and discharged by 30 June 2020 (ii) the control group were admitted between 1 March 2019 to 1 June 2019, and discharged by 30 June 2019. The pandemic was declared by the World Health Organisation on the 11th of March 2019, but in the days before this there was an increase in the number of reported infections in the area served by our institution, so the study period was chosen from the 1st March 2019. Excluded were all patients that did not have one of the ICD-10 codes above. Mortality was established using NHS Digital data.

Demographic and injury data collected included patient age and sex, the diagnosis, COVID status (as identified in the medical records as "Coronavirus SARS-COV-2" positive patients or with a positive reverse transcriptase polymerase chain reaction; rPCR), number of comorbidities and whether or not the patient had a surgical procedure (as identified through the procedure codes of the submitted Hospital Episode Statistics data; HES). For the subgroup of hip fracture patients, prospectively collected data was obtained from the institutional local hip fracture database. On this database, data for every adult patient admitted with a hip fracture is recorded using a modified version of the Standardised Audit of Hip Fractures in Europe (SAHFE) data collection form. Data analysed included mechanism of injury, hip fracture type, body mass index (BMI), abbreviated mental test score (AMTS,[8]), mobility status (Fracture Mobility Score [9]), Nottingham hip fracture score [10], haemoglobin concentration (Hb, as measured from the full blood count blood test) on admission and clinical frailty (as recorded on the Rockwood Clinical Frailty Scale; RCFS [11]). Data collected was confidential and managed in line with national data protection guidelines. Hospital outcome data were available for all patients and included type of surgery, mortality, requirement for invasive or non-invasive ventilation, length of stay and development of complications.

The data was extracted by a data analyst from our local database in July 2020 and ordered in Microsoft Excel (Redmond, WA, US) where numerical data comparison between groups was carried out using a student t-test assuming a 2-tailed distribution with homoscedastic variance. For categorical data, where a 2x2 contingency table could be used, probability was calculated with the Fisher's exact test. For contingency tables greater than 2x2, the Chi-squared test was used. Statistical significance was set at $p<0.05$. Mortality was calculated from any death of any member of the cohort between 1st March and the 29th June in the respective year. Cox proportional hazards model were calculated using STATA 16.0 (StataCorp LLC, Texas, US) statistical software package, to compare survival between groups and to calculate the Hazard Ratio (HR) where the COVID 1st wave was the covariate. In addition, Student t-test (assuming a 2-tailed distribution with homoscedastic variance), Fisher exact test and Chi-squared test were used to calculate probability. The study was part of an approved local hospital audit (no. 20-333-C).

<u>Patient and public involvement</u>

Patients or the public were not involved in the design, or conduct, or reporting, or dissemination plans of our research.

For peer review only - http://bmjopen.bmj.com/site/about/guidelines.xhtml

Results

Demographic comparison between groups is shown in Table 1a. The percentage female and the number of hip fractures were equivalent between groups. Differences were noted with age and a lower percentage of patients receiving operative treatment during the COVID-19.

Hip fractures were the most common diagnoses making up approximately 20% in each of the two comparative groups. These were selected for a more granular subgroup analysis. Table 1b shows the variables used to assess selection bias between the hip fracture subgroups. No differences were seen between subgroups for the variables selected other than prevalence of COVID-19 infection.

Outcomes are recorded in Table 2, showing that mortality was significantly higher in the cohort presenting with a fragility fracture during the COVID-19 pandemic period. This was seen both in the COVID-19 period group as a whole and in the hip fracture sub-group analysis. It was also noted that the length of stay was significantly shorter for the COVID-19 period group and the COVID-19 period hip fracture subgroup.

Mortality of fragility fracture patients without COVID-19 was significantly higher among pandemic period admissions (14.7%) than in the pre-pandemic cohort (10.2%) after adjusting for age and sex (hazards ratio (HR)=1.86; 95%CI 1.41-2.45; p<0.0001) (Figures 1 and 2).

Table 3a shows variables that may have influenced outcomes. The cohort of COVID-19 positive patients was significantly older than the COVID-19 negative patients in the same time period. In relation to excess deaths in non-COVID patients, Table 3a also shows that the COVID-19 negative patients had a significantly shorter length of stay, lower frequency of operative treatment and less than expected ventilatory support than controls. There were no differences seen for sex or age between groups.

A sub-analysis of hip fracture patients revealed a mortality of 8.4% among 190 admissions in the pre-pandemic set, and of 15.48% among 168 pandemic admissions with no COVID diagnosis, resulting in a HR=2.08 95%CI 1.11-3.90 p=0.021. After further adjustment for clinical frailty scores this became HR=2.15 95%CI 1.15-4.04 p=0.0162.

Discussion

In this study we report significantly higher mortality among fragility fracture patients with no COVID-19 related diagnosis admitted during the COVID-19 pandemic period compared to patients admitted with the same ICD-10 codes to the same hospital in the same time period of the year 2019. We also report significantly shorter hospital length of stay during the months of the pandemic compared to the same period a year earlier. These results hold true after adjustment for potential confounders such as age and sex. A sub-analysis on femoral neck fractures where frailty indices and other clinical assessments were readily available showed a similar pattern whilst there was no difference in frailty between the pandemic and pre-pandemic cohorts.

For peer review only - http://bmjopen.bmj.com/site/about/guidelines.xhtml

Limitations of this study include potential variables that have not been controlled that may have influenced the effects that have been seen. Knowledge of the fragility fracture patients that contracted COVID-19 in the community, who subsequently died within the period of assessment are not known and may account for a proportion of the excess mortality. In addition, it is known that during the first-wave period of investigation in this study, the institutional compliance with the best practice tariff was reduced. There is the potential that the redeployment of staff during the COVID-19 first wave, particularly the orthogeriatric support and physiotherapists, for fragility fracture patients, may have had a negative influence on outcomes. However, specific data about the effect of redeployment as a variable is not available for this study.

A recent report from the Office for National Statistics found that England had the highest overall relative excess mortality out of all the European countries compared during the first 6 months of 2020 [12]. While none of the four UK nations had a peak mortality level as high as Spain or the worst-hit local areas of Spain and Italy, excess mortality was geographically widespread throughout the UK during the pandemic, whereas it was more geographically localised in most countries of Western Europe. Our data add to this picture by showing that excess mortality involves patients without COVID-19 and areas of England with rates of infection below the national average. Achievement of best practice hip fracture tariff [13] at our institution during the pandemic fell from an average of 42% (Mar-Jun 2019) to 38% (Mar-Jun 2020) with a particular failure to achieve the delirium assessment, which fell from an average of 96% to 62% for the respective periods. In addition, there was a significant decrease in the number of hip fracture patients who were not delirious after surgery, from 81% to 63% respectively. This indicates breakdown of standard care pathways due to extrinsic pressures and provides a likely explanation for the excess mortality. As there are no agreed pathways or metrics for other fractures it is impossible to prove, although probable that the same factors apply.

Hip fractures occurred at the same frequency during the COVID-19 period and the national lockdown, as before it, with no differences identified for age, sex, BMI, AMTS, mobility, NHFS, anaemia, fracture type or frailty between groups. Similar incidence pre- and post- COVID with similar demographics allows a valid comparison to examine excess deaths.

Analysis of the COVID-19 negative subset of those presenting during the COVID-19 period compared to the control period does identify associations that may be relevant. Whilst there were no differences identified between these groups for age, the development of ARDS or sepsis of the requirement for ventilatory support post procedure, significant difference was noted in the COVID-19 period group (COVID-19 negative subset) for a shorter length of stay and reduced number of procedures. Both differences suggest that the standard care pathways were altered for fragility fractures, which is further supported by the observed fall in BPT achievement. A reduced length of stay is often considered a positive outcome. However, if the threshold for a safe discharge is altered, for instance in the face of a great need for more inpatient beds to manage a pandemic, there may be unintended consequences affecting mortality. A significant proportion of patients with fragility fractures require input from health and social care in the form of rehab services and residential homes. During the pandemic

For peer review only - http://bmjopen.bmj.com/site/about/guidelines.xhtml

period it was unknown regarding the virulence of spread of Covid-19 transmission within care homes and limiting testing in this group of people was available, this may have also influenced our mortality findings.

For those presenting with hip fractures during the COVID-19 period, who did not test positive for COVID-19, that no differences were seen for age, sex, BMI, treatment type (including non-operative treatment) and complications including: venous thromboembolism, urinary tract infection, stroke, myocardial infarction, failure of fixation or dislocation, transfusion, acute renal failure, pulmonary embolus or clostridium difficile when compared to the control group suggests that understanding the reasons for the excess mortality is complex. Despite this, reduction in length of stay seems to be a significant factor in the hip fracture subgroup analysis, where changes to operative treatment frequency (as for the fragility fracture group as a whole) is not. It has been demonstrated that a COVID-19 infection independently causes an increased mortality compared to those presenting with hip fractures that do not contract COVID-19 [14-16].

During the COVID-19 period, only 12 patients tested positive for COVID-19. When compared to those presenting during the same time period, they were older and had a longer length of stay. Age, as a risk factor for developing COVID-19, is well established [17].

These data suggest that excess deaths are likely due to changing established, evidence-based care-pathways, particularly regarding thresholds for safe discharge and length of stay. Excess mortality seen in this vulnerable fragility fracture population is likely to be minimised by maintaining standards of care during further waves of the COVID-19 or future pandemics.

For peer review only

References

1. ONS Deaths involving COVID-19, England and Wales - Office for National Statistics. https://www.ons.gov.uk/peoplepopulationandcommunity/birthsdeathsandmarriages/deaths/bulletins/deathsinvolvingcovid19englandandwales/deathsoccurringinjune2020 (accessed 27/07/2020).

2. Kluge, H. Statement – Older people are at highest risk from COVID-19, but all must act to prevent community spread. https://www.euro.who.int/en/health-topics/health-emergencies/coronavirus-covid-19/statements/statement-older-people-are-at-highest-risk-from-covid-19,-but-all-must-act-to-prevent-community-spread. (accessed 27/07/2020)

3. Verity, R.; Okell, L. C.; Dorigatti, I.; Winskill, P.; Whittaker, C.; Imai, N.; Cuomo-Dannenburg, G.; Thompson, H.; Walker, P. G. T.; Fu, H.; Dighe, A.; Griffin, J. T.; Baguelin, M.; Bhatia, S.; Boonyasiri, A.; Cori, A.; Cucunubá, Z.; FitzJohn, R.; Gaythorpe, K.; Green, W.; Hamlet, A.; Hinsley, W.; Laydon, D.; Nedjati-Gilani, G.; Riley, S.; van Elsland, S.; Volz, E.; Wang, H.; Wang, Y.; Xi, X.; Donnelly, C. A.; Ghani, A. C.; Ferguson, N. M., (2020) Estimates of the severity of coronavirus disease 2019: a model-based analysis. *Lancet Infect Dis*, ***20*** (6), 669-677.

4. Piccininni, M.; Rohmann, J. L.; Foresti, L.; Lurani, C.; Kurth, T., (2020) Use of all cause mortality to quantify the consequences of covid-19 in Nembro, Lombardy: descriptive study. *Bmj*, ***369***, m1835.

5. Buss, L.; McKeever, T. M.; Nightingale, J.; Akyea, R.; Ollivere, B.; Moppett, I. K.; Bolton, C. E., (2018) Hip fracture outcomes in patients with chronic obstructive pulmonary disease. *Br J Anaesth*, ***121*** (6), 1377-1379.

6. Ollivere, B.; Rollins, K.; Brankin, R.; Wood, M.; Brammar, T. J.; Wimhurst, J., (2012) Optimising fast track care for proximal femoral fracture patients using modified early warning score. *Ann R Coll Surg Engl*, ***94*** (4), 267-71.

7. World Health Organization: (1992) *The ICD-10 classification of mental and behavioural disorders : clinical descriptions and diagnostic guidelines*.

8. Hodkinson, H., (1972) Evaluation of a mental test score for assessment of mental impairment in the elderly. *Age and ageing*, ***1*** (4), 233-238.

9. Voeten, S. C.; Nijmeijer, W. S.; Vermeer, M.; Schipper, I. B.; Hegeman, J. H.; group, D. T. s., (2020) Validation of the Fracture Mobility Score against the Parker Mobility Score in hip fracture patients. *Injury*, ***51*** (2), 395-399.

10. Maxwell, M. J.; Moran, C. G.; Moppett, I. K., (2008) Development and validation of a preoperative scoring system to predict 30 day mortality in patients undergoing hip fracture surgery. *Br J Anaesth*, ***101*** (4), 511-7.

11. Rockwood, K.; Song, X.; MacKnight, C.; Bergman, H.; Hogan, D. B.; McDowell, I.; Mitnitski, A., (2005) A global clinical measure of fitness and frailty in elderly people. *Cmaj*, ***173*** (5), 489-95.

12. ONS, O. f. N. S. Comparisons of all-cause mortality between European countries and regions: January to June 2020 https://www.ons.gov.uk/peoplepopulationandcommunity/birthsdeathsandmarriages/deaths/articles/comparisonsofallcausemortalitybetweeneuropeancountriesandregions/januarytojune2020 (accessed 25/08/2020).

13. Royal-College-of-Physicians National Hip Fracture Database - Charts & Reports. https://www.nhfd.co.uk/20/NHFDCharts.nsf. (accessed 25/08/2020)

For peer review only - http://bmjopen.bmj.com/site/about/guidelines.xhtml

14. Kayani, B.; Onochie, E.; Patil, V.; Begum, F.; Cuthbert, R.; Ferguson, D.; Bhamra, J. S.; Sharma, A.; Bates, P.; Haddad, F. S., (2020) The effects of COVID-19 on perioperative morbidity and mortality in patients with hip fractures. *Bone Joint J*, *102-B* (9), 1136-1145.

15. Arafa, M.; Nesar, S.; Abu-Jabeh, H.; Jayme, M. O. R.; Kalairajah, Y., (2020) COVID-19 pandemic and hip fractures: impact and lessons learned. *Bone Jt Open*, *1* (9), 530-540.

16. Clement, N. D.; Ng, N.; Simpson, C. J.; Patton, R. F. L.; Hall, A. J.; Simpson, A. H. R. W.; Duckworth, A. D., (2020) The prevalence, mortality, and associated risk factors for developing COVID-19 in hip fracture patients: a systematic review and meta-analysis. *Bone Joint Res*, *9* (12), 873-883.

17. Li, X.; Xu, S.; Yu, M.; Wang, K.; Tao, Y.; Zhou, Y.; Shi, J.; Zhou, M.; Wu, B.; Yang, Z.; Zhang, C.; Yue, J.; Zhang, Z.; Renz, H.; Liu, X.; Xie, J.; Xie, M.; Zhao, J., (2020) Risk factors for severity and mortality in adult COVID-19 inpatients in Wuhan. *J Allergy Clin Immunol*, *146* (1), 110-118.

For peer review only

For peer review only - http://bmjopen.bmj.com/site/about/guidelines.xhtml

a.			
**Factor**	**COVID-19 period group (admitted between 01/03/2020 and 01/06/2020) (n= 832)**	**Control period group (admitted between 01/03/2019 and 01/06/2019) (n= 1014)**	**p value**
Mean age, years (± SD)	74.8 (± 20.0)	72.8 (± 21.4)	0.045[1]
Female, n (%)	492/832 (59.1%)	629/1014 (62.0%)	0.213[2]
rPCR COVID-19 positive, n (%)	104/832 (12.5%)	0/1014 (0%)	<0.00001[2]
Undergoing operative procedure, n (%)	70/832 (8.4%)	765/1014 (75.4%)	<0.00001[2]
Hip fracture, n (%)	180/832 (21.6%)	190/1014 (18.7%)	0.129[2]
**b.**			
**Factor**	**COVID-19 period hip fracture subgroup (total: n= 180)**	**Control period hip fracture subgroup (total: n= 190)**	**p value**
Mean age, years (± SD)	83.2 (± 8.8)	81.9 (± 9.2)	0.183[1]
Female, n (%)	132/180 (73.3%)	139/190 (73.2%)	1.000[2]
rPCR Covid positive, n (%)	12/180 (6.7%)	0/190 (0%)	<0.0001[2]
Mean BMI, Kg/m^2 (± SD); n	24.2 (± 5.2); 159	23.2 (± 4.4); 169	0.070[1]
Mean AMTS, score (± SD); n	6.9 (± 3.5); 179	6.7 (± 3.9); 189	0.538[1]
Mobility status, n (available; %)	n=175	n=187	0.076[3]
• Freely mobile, no aids	61/175 (34.9%)	75/187 (40.1%)	
• Outside with aids	107/175 (61.1%)	96/187 (51.3%)	
Unable to mobilise outside	7/175 (4.0%)	16/187 (8.6%)	
Mean Nottingham Hip fracture score (± SD); n	0.082 (±0.063); 172	0.081 (± 0.054); 181	0.838[1]
Anaemia on admission, Hb <12 g/L, n (%)	36/175 (20.6%)	45/182 (24.7%)	0.378[2]
Hip fracture type			
• Undisplaced intracapsular, n (%)	14/180 (7.8%)	16/190 (8.4%)	0.851[2]
• Displaced intracapsular, n (%)	76/180 (42.2%)	83/190 (43.7%)	0.398[2]
• Intertrochanteric, n (%)	77/180 (42.8%)	79/190 (41.6%)	0.834[2]
Reverse oblique or subtrochanteric, n (%)	13/180 (7.2%)	12/190 (6.3%)	0.837[2]

For peer review only

RCFS, mean category from 1-9 (± SD); n	4.8 (± 1.6); 155	4.6 (± 1.8); 175	0.421[1]

Table 1 a. Descriptive characteristics of all fragility fracture patients presenting during the two study periods. b. Hip fracture subgroup analysis for the two study periods rPCR (reverse transcriptase polymer chain reaction), BMI (body mass index), AMTS (abbreviated mental test score), Hb (whole blood haemoglobin), RCFS (Rockwood Clinical Frailty Scale); [1]Student t-test assuming a 2-tailed distribution with homoscedastic variance. [2]Fisher exact test. [3]Chi-squared statistic calculated on a 2x3 contingency table.

For peer review only

**a. Outcomes**	**COVID-19 period group (n= 832)**	**Control group (n= 1014)**	**Hazard Ratio (HR) and p value**
Mortality, n (%)	150/832 (18.0%)	104/1014 (10.2%)	HR 1.86; 95%CI 1.41-2.45; p<0.0001
Length of stay, mean days (± SD); n	8.8 (± 8.9), 832	12.1 (14.3), 1014	p<0.00011
**b. Outcomes**	**COVID-19 period hip fracture sub-group (n=168)**	**Control hip fracture subgroup (n=190)**	**Hazard Ratio (HR) and p value**
Mortality, n (%)	30/180 (15.5%)	16/190 (8.4%)	HR=2.08; 95%CI 1.11-3.90 p=0.021
Mortality adjusted for clinical frailty, n (%)	30/180 (15.5%)	16/190 (8.4%)	HR=2.15; 95%CI 1.15-4.04 p=0.0162
Length of stay, mean days, (± SD); n	11.1 (± 5.5); 180	15.3 (± 7.4); 190	p<0.0001[1]

Table 2. Outcomes for each group. a. Outcomes for the full cohort. b. Outcomes for the hip fracture subgroup. SD (Standard Deviation), HR (Hazard Ratio), n (number). [1]2-tailed distribution with homoscedastic variance, Student's t-test.

a.	COVID period group COVID-19 positive (total: n= 104)	COVID period group COVID-19 negative (total: n=728)	Control period group (total: n= 1014)	p value
Age on admission; mean years (±SD); n	81.0 (±11.9)	73.9 (±20.7)		0.001[1]
		73.9 (±20.7)	72.8 (± 21.4)	0.301[1]
Male sex; n (%)	47/104 (45.2%)	292/728 (40.1%)		0.338[2]
		292/728 (40.1%)	385/1014 (38.0%)	0.370[2]
Length of stay; mean days (± SD); n	14.8 (±10.6)	8.0 (±8.3)		<0.00001[1]
		8.0 (±8.3)	12.1 (±14.3)	<0.00001[1]
Operative treatment; n (%)	12/104 (11.5%)	58/728 (8.0%)		0.255[2]
		58/728 (8.0%)	765/1014 (75.4%)	<0.00001[2]
ARDS or sepsis, n (%)	5/104 (4.8%)	26/728 (3.6%)		0.576[2]
		26/728 (3.6%)	26/1014 (2.6%)	0.254[2]
Ventilation post procedure, n (%)	6/104 (5.8%)	24/728 (3.3%)		0.253[2]
		24/728 (3.3%)	54/1014 (5.3%)	0.046[2]

b.	COVID period hip fracture subgroup COVID-19 positive (total: n= 12)	COVID period hip fracture subgroup COVID-19 negative (total: n= 168)	Control hip fracture subgroup (total: n= 190)	p value

For peer review only

Age on admission; mean years (±SD); n	82.5 (±6.3)	83.1 (±9.0)		0.818[1]
		83.1 (±9.0)	81.9 (± 9.2)	0.181[2]
Male sex; n (%)	4/12	44/168 (26.1%)		0.736[2]
		44/168 (26.1%)	51/190 (26.8)	0.905[2]
Length of stay; mean days (± SD); n	17.3 (±7.2)	10.7 (±5.15)		<0.00001[1]
		10.7 (±5.15)	15.3 (± 7.4)	<0.00001[2]
Hip fracture treatment				
• Arthroplasty	8/12	68/165* (41.2%)	81/190 (42.6%)	0.227[3]
• Fixation	4/12	90/165* (54.6%)	103/190 (54.2%)	0.355[3]
• Non-operative	0/12	7/165* (4.2%)	6/190 (3.2%)	0.587[3]
Pulmonary embolus or DVT	0/12	0/168	2/190 (1.1%)	
Pneumonia	3/12	14/168 (8.3%)	13/190 (6.8%)	0.081[3]
UTI	3/12	7/168 (4.2%)		0.021[2]
		7/168 (4.2%)	10/190 (5.3%)	0.804[2]
CVA	0/12	0/168	0/190	
MI or ACS	0/12	2/168 (1.2%)	0/190	
Dislocation or failure of fixation	0/12	0/168	1/190 (0.5%)	
Blood transfusion	2/12	32/168 (19.1%)	40/190 (21.1%)	0.856[3]
Renal failure	2/12	18/168 (10.7%)	22/190 (11.6%)	0.813[3]
Pressure ulcer	0/12	1/168 (0.6%)	1/190 (0.5%)	
Clostridium difficile	0/12	0/168	0/190	
BMI	24.7 (±3.5); 9	24.27 (±5.1);150		0.768[1]
		24.27 (±5.1);150	23.2 (± 4.4); 169	0.072[2]

Table 3. Complications, patient and treatment factors by group. [1]2-tailed distribution with homoscedastic variance Student's t-test. [2]Fisher exact test. [3] Chi-squared statistic calculated on a 2x3 contingency table.* Data was only available for 165 patients.

For peer review only

For peer review only - http://bmjopen.bmj.com/site/about/guidelines.xhtml

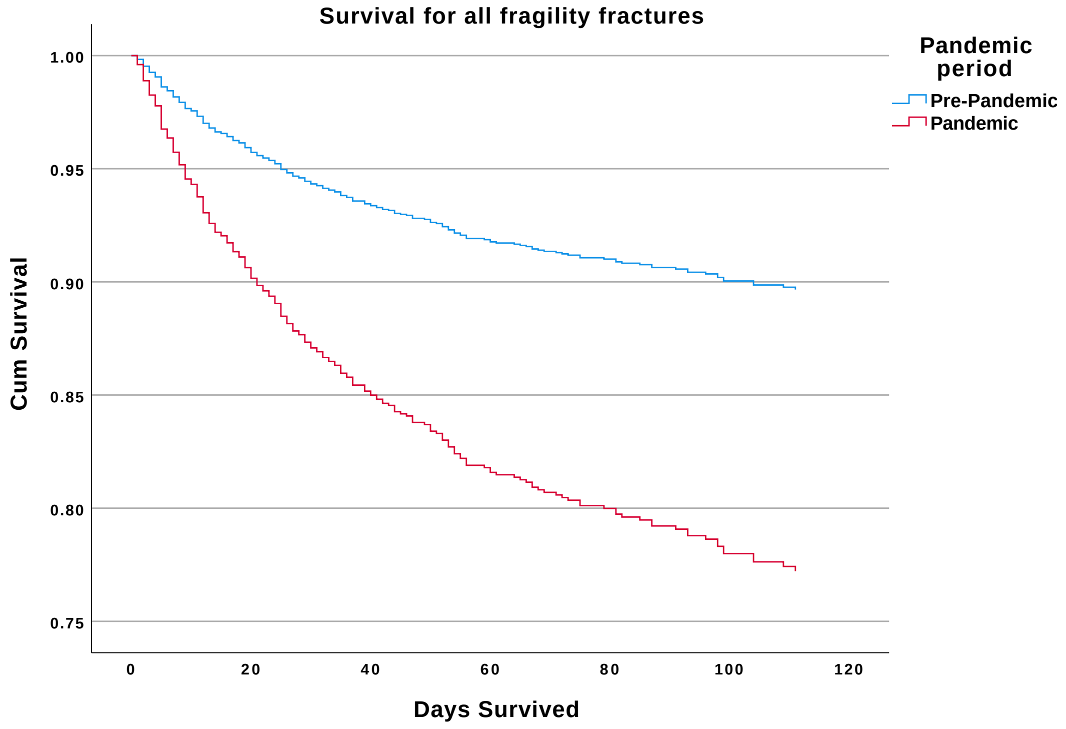

For peer review only - http://bmjopen.bmj.com/site/about/guidelines.xhtml

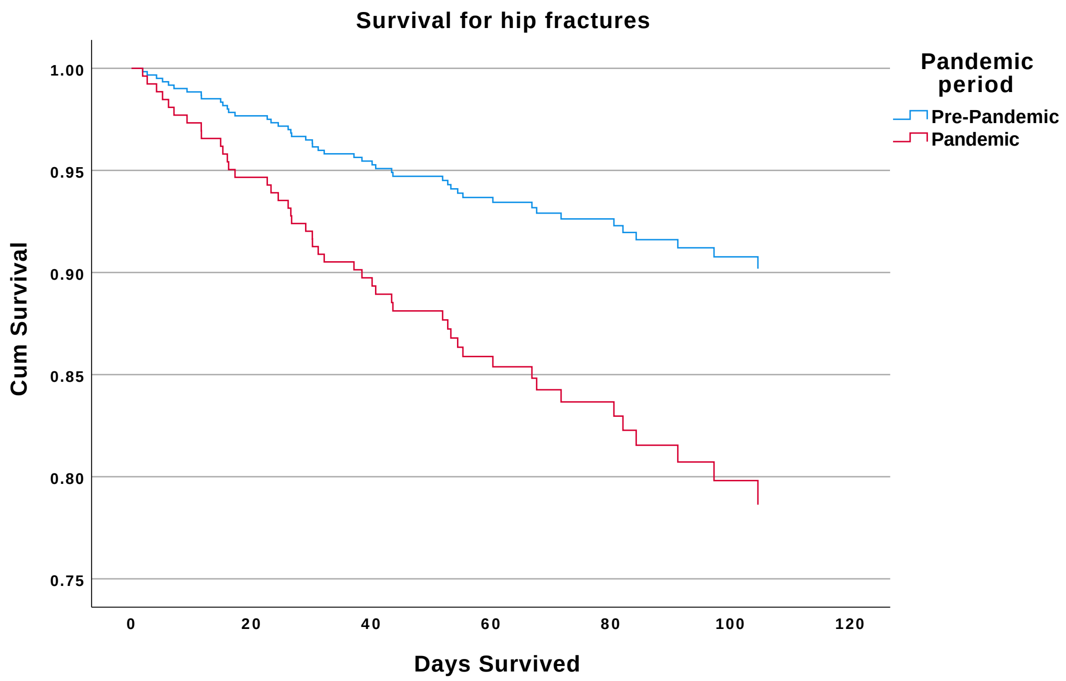

For peer review only

For peer review only - http://bmjopen.bmj.com/site/about/guidelines.xhtml

STROBE Statement—Checklist of items that should be included in reports of ***cohort studies***

	Item No	Recommendation	Page No
**Title and abstract**	1	(*a*) Indicate the study's design with a commonly used term in the title or the abstract	1
		(*b*) Provide in the abstract an informative and balanced summary of what was done and what was found	3
**Introduction**			
Background/rationale	2	Explain the scientific background and rationale for the investigation being reported	6
Objectives	3	State specific objectives, including any prespecified hypotheses	7
**Methods**			
Study design	4	Present key elements of study design early in the paper	7
Setting	5	Describe the setting, locations, and relevant dates, including periods of recruitment, exposure, follow-up, and data collection	7
Participants	6	(*a*) Give the eligibility criteria, and the sources and methods of selection of participants. Describe methods of follow-up<br>(*b*) For matched studies, give matching criteria and number of exposed and unexposed	7-8
Variables	7	Clearly define all outcomes, exposures, predictors, potential confounders, and effect modifiers. Give diagnostic criteria, if applicable	8
Data sources/ measurement	8*	For each variable of interest, give sources of data and details of methods of assessment (measurement). Describe comparability of assessment methods if there is more than one group	7-8
Bias	9	Describe any efforts to address potential sources of bias	9
Study size	10	Explain how the study size was arrived at	7
Quantitative variables	11	Explain how quantitative variables were handled in the analyses. If applicable, describe which groupings were chosen and why	8
Statistical methods	12	(*a*) Describe all statistical methods, including those used to control for confounding<br>(*b*) Describe any methods used to examine subgroups and interactions<br>(*c*) Explain how missing data were addressed<br>(*d*) If applicable, explain how loss to follow-up was addressed<br>(*e*) Describe any sensitivity analyses	8-9
**Results**			
Participants	13*	(a) Report numbers of individuals at each stage of study—eg numbers potentially eligible, examined for eligibility, confirmed eligible, included in the study, completing follow-up, and analysed<br>(b) Give reasons for non-participation at each stage<br>(c) Consider use of a flow diagram	9, 16-21
Descriptive data	14*	(a) Give characteristics of study participants (eg demographic, clinical, social) and information on exposures and potential confounders<br>(b) Indicate number of participants with missing data for each variable of interest<br>(c) Summarise follow-up time (eg, average and total amount)	9, 16-21
Outcome data	15*	Report numbers of outcome events or summary measures over time	9, 16-21

For peer review only - http://bmjopen.bmj.com/site/about/guidelines.xhtml

For peer review only

For peer review only - http://bmjopen.bmj.com/site/about/guidelines.xhtml

Main results	16	(*a*) Give unadjusted estimates and, if applicable, confounder-adjusted estimates and their precision (eg, 95% confidence interval). Make clear which confounders were adjusted for and why they were included<br>(*b*) Report category boundaries when continuous variables were categorized<br>(*c*) If relevant, consider translating estimates of relative risk into absolute risk for a meaningful time period	9,15-20
Other analyses	17	Report other analyses done—eg analyses of subgroups and interactions, and sensitivity analyses	9, 15-20
**Discussion**			
Key results	18	Summarise key results with reference to study objectives	10-12
Limitations	19	Discuss limitations of the study, taking into account sources of potential bias or imprecision. Discuss both direction and magnitude of any potential bias	10
Interpretation	20	Give a cautious overall interpretation of results considering objectives, limitations, multiplicity of analyses, results from similar studies, and other relevant evidence	10-12
Generalisability	21	Discuss the generalisability (external validity) of the study results	11-12
**Other information**			
Funding	22	Give the source of funding and the role of the funders for the present study and, if applicable, for the original study on which the present article is based	2

*Give information separately for exposed and unexposed groups.

Note: An Explanation and Elaboration article discusses each checklist item and gives methodological background and published examples of transparent reporting. The STROBE checklist is best used in conjunction with this article (freely available on the Web sites of PLoS Medicine at http://www.plosmedicine.org/, Annals of Internal Medicine at http://www.annals.org/, and Epidemiology at http://www.epidem.com/). Information on the STROBE Initiative is available at http://www.strobe-statement.org.

For peer review only

For peer review only - http://bmjopen.bmj.com/site/about/guidelines.xhtml

BMJ Open

Has a change in established care pathways during the 1st wave of the Covid-19 pandemic led to an excess death rate in the fragility fracture population? A longitudinal cohort study of 1846 patients.

Journal:	*BMJ Open*
Manuscript ID	bmjopen-2021-058526.R1
Article Type:	Original research
Date Submitted by the Author:	10-Mar-2022
Complete List of Authors:	Ikram, Adeel; University of Nottingham School of Medicine, Trauma & Orthopaedics; Nottingham University Hospitals NHS Trust<br>Norrish, Alan; University of Nottingham School of Medicine, Trauma and Orthopaedics; Queen Elizabeth Hospital King's Lynn NHS Foundation Trust<br>Ollivere, Luke; Nottingham University Hospitals NHS Trust<br>Nightingale, Jessica; University of Nottingham School of Medicine, Trauma and Orthopaedics; Nottingham University Hospitals NHS Trust<br>Valdes, A; University of Nottingham School of Medicine; NIHR Nottingham Biomedical Research Centre<br>Ollivere, Benjamin; University of Nottingham School of Medicine, Trauma & Orthopaedics; Nottingham University Hospitals NHS Trust
**Primary Subject Heading**:	Surgery
Secondary Subject Heading:	Health policy, Infectious diseases, Public health, Evidence based practice
Keywords:	COVID-19, ORTHOPAEDIC & TRAUMA SURGERY, Hip < ORTHOPAEDIC & TRAUMA SURGERY, Health policy < HEALTH SERVICES ADMINISTRATION & MANAGEMENT, Organisation of health services < HEALTH SERVICES ADMINISTRATION & MANAGEMENT, Risk management < HEALTH SERVICES ADMINISTRATION & MANAGEMENT

SCHOLARONE™
Manuscripts

I, the Submitting Author has the right to grant and does grant on behalf of all authors of the Work (as defined in the below author licence), an exclusive licence and/or a non-exclusive licence for contributions from authors who are: i) UK Crown employees; ii) where BMJ has agreed a CC-BY licence shall apply, and/or iii) in accordance with the terms applicable for US Federal Government officers or employees acting as part of their official duties; on a worldwide, perpetual, irrevocable, royalty-free basis to BMJ Publishing Group Ltd ("BMJ") its licensees and where the relevant Journal is co-owned by BMJ to the co-owners of the Journal, to publish the Work in this journal and any other BMJ products and to exploit all rights, as set out in our licence.

The Submitting Author accepts and understands that any supply made under these terms is made by BMJ to the Submitting Author unless you are acting as an employee on behalf of your employer or a postgraduate student of an affiliated institution which is paying any applicable article publishing charge ("APC") for Open Access articles. Where the Submitting Author wishes to make the Work available on an Open Access basis (and intends to pay the relevant APC), the terms of reuse of such Open Access shall be governed by a Creative Commons licence – details of these licences and which Creative Commons licence will apply to this Work are set out in our licence referred to above.

Other than as permitted in any relevant BMJ Author's Self Archiving Policies, I confirm this Work has not been accepted for publication elsewhere, is not being considered for publication elsewhere and does not duplicate material already published. I confirm all authors consent to publication of this Work and authorise the granting of this licence.

For peer review only

Has a change in established care pathways during the 1st wave of the Covid-19 pandemic led to an excess death rate in the fragility fracture population? A longitudinal cohort study of 1846 patients.

Adeel Ikram[1,2], Alan R Norrish [1,2,4], Luke Ollivere[2], Jessica Nightingale[1,2], Ana M Valdes[1,3], Benjamin J Ollivere[1,2,3]

Affiliations

[1] School of Medicine, University of Nottingham

[2] Nottingham University Hospitals NHS Trust

[3] NIHR Nottingham Biomedical Research Unit (BRC), UK

[4] Queen Elizabeth Hospital, King's Lynn, UK

Corresponding Author:

Mr Adeel Ikram

Specialty Registrar Trauma & Orthopaedics

University of Nottingham Queen's Medical Centre,

Academic Orthopaedics, C Floor, West Block

Nottingham University Hospitals NHS Trust, Derby Road,

Nottingham, NG7 2UH

Email: adeel.ikram@nottingham.ac.uk

Word Count (Excluding title page and references): 3311

Tables: 3

Figures: 2

Key Words: COVID-19, excess death, fragility fracture, hip fracture

Declarations

Ethical Approval –. The study was registered and approved with our local hospital audit department (no. 20-333-C). This study did not require ethical approval, we used anonymised data that was collected as part of routine care for clinical audit.

Consent to participate and consent to publish – Consent to participate was not required as this was an analysis of anonymised patient data collected as part of routine care for clinical audit.

Authors Contributions

Adeel Ikram ORCID - 0000-0002-7520-6949

Methodology, data analysis and interpretation, original draft preparation, review, editing and revision of manuscript.

Alan Norrish ORCID - 0000-0003-3735-1042

For peer review only

Methodology, data analysis and interpretation, original draft preparation review and editing.

Luke Ollivere

Hospital coding database manager, data extraction and acquisition, resources.

Jessica Nightingale ORCID – 0000-0001-8359-5705

Database manager, data extraction and acquisition, resources, reviewed manuscript.

Ana Valdes ORCID- 0000-0003-1141-4471

Conceptualised study, methodology, secured funding, interpretation of data, reviewed manuscript, approved version to be published. Supervised study

Benjamin Ollivere ORCID - 0000-0002-1410-1756

Conceptualised study, methodology, interpretation of data, reviewed manuscript, approved version to be published. Supervised study.

Funding – The research leading to these results received funding from UKRI/MRC Rapid Response COVID trial under grant number (MR/V027883/1).

Conflicts of interest/ Competing interests

- The authors have no relevant financial or non-financial interests to disclose.
- The authors have no conflicts of interest to declare that are relevant to the content of this article.
- All authors certify that they have no affiliations with or involvement in any organisation or entity with any financial interest or non-financial interest in the subject matter or materials discussed in this manuscript.
- The authors have no financial or proprietary interests in any material discussed in this article.

Availability of data and materials

The datasets generated during and/or analysed during the current study are not publicly available due to them containing patient identifiable data within the NHS. But anonymised data are available from the corresponding author on reasonable request.

For peer review only

Has a change in established care pathways during the 1st wave of the Covid-19 pandemic led to an excess death rate in the fragility fracture population? A longitudinal cohort study of 1846 patients.

Abstract

Objective

During the 1st wave of the COVID-19 pandemic, changes to established care pathways and discharge thresholds for patients with fragility fractures were made. This was to increase hospital bed capacity and minimise the inpatient risk of contracting COVID-19. This study aims to identify the excess death rate in this population during the first wave of the pandemic.

Design

A longitudinal cohort study of patients with fragility fractures identified by specific ICD-10 codes. The first wave of the pandemic was defined as the 3-month period between 1st March to 1st June 2020. The control group presenting between 1st March to 1st June 2019.

Setting

Two acute National Health Service (NHS) Hospitals within the East Midlands region of England.

Participants

1846 patients with fragility fractures over the aforementioned two specified matched time points.

Primary and Secondary outcome measures

Four-month mortality of all patients with fragility fractures with a sub analysis of patients with fragility hip fractures.

Results

832 patients with fragility fracture were admitted during the pandemic period (104 diagnosed with COVID-19). 1014 patients presenting with fragility fractures in the control group. Mortality in fragility fracture patients without COVID-19 were significantly higher amongst pandemic period admissions (14.7%) than the pre-pandemic cohort (10.2%), (Hazards-Ratio (HR)=1.86; 95%CI 1.41-2.45; p<0.0001) adjusted for age and sex. Length of stay was shorter during the pandemic period (effect size =-4.2 days 95%CI -5.8, -3.1, p<0.0001). Sub-analysis of fragility hip fracture patients revealed a mortality of 8.4% in the pre-pandemic cohort, and 15.48% during pandemic admissions with no COVID-19 diagnosis, HR=2.08 95%CI 1.11-3.90 p=0.021.

Conclusions

There is a significant increase in excess death, not explained by confirmed COVID-19 infections. Altered care-pathways and aggressive discharge criteria during the pandemic are likely responsible for the increase in excess deaths.

For peer review only

Article Summary

Strengths and limitations of this study

- This study is one of the largest cohort studies in fragility hip fracture patients investigating the excess mortality in Covid-19 negative patients during the 1st wave of the Covid-19 pandemic.
- Specific ICD-10 codes allowed us to identify the same injury patterns over both groups and a comparative time matched cohort was used as a control.
- The effect of the provision of personal protective equipment and transmission between healthcare staff and patients was not known and this data was not available.
- The redeployment of support staff particularly physiotherapists and orthogeriatric staff for fragility fracture patients may have had an influence on negative outcomes, however specific redeployment data was not available for this study.

Funding

The research leading to these results received funding from UKRI/MRC Rapid Response COVID trial under grant number (MR/V027883/1).

Conflicts of interest/ Competing interests

- The authors have no relevant financial or non-financial interests to disclose.
- The authors have no conflicts of interest to declare that are relevant to the content of this article.
- All authors certify that they have no affiliations with or involvement in any organisation or entity with any financial interest or non-financial interest in the subject matter or materials discussed in this manuscript.
- The authors have no financial or proprietary interests in any material discussed in this article.

Availability of data and materials

The datasets generated and/or analysed during the current study are not publicly available due to them containing patient identifiable data within the NHS. But anonymised data are available from the corresponding author upon reasonable request.

For peer review only - http://bmjopen.bmj.com/site/about/guidelines.xhtml

Introduction

The Office of National Statistics in the United Kingdom, states between the 1st March and 30th June 2020 there were 218,837 deaths in England and Wales, of which 50,335 (23%) involved the coronavirus (COVID-19) [1]. At the start of March 2020, the number of deaths per day was below the five-year average, possibly due to mild winter levels of circulating influenza. By the end of the first week of April 2020 this figure was more than double the five-year average, demonstrating the significant impact of COVID-19 on all-cause mortality [1].

The first wave of the COVID-19 pandemic transformed the delivery of orthopaedic services. There was cessation of elective operative practice, a reduction in trauma operative capacity as intensive care bed capacity needed to expand into theatres, and redeployment of all grades of orthopaedic staff (surgeons, medics and allied healthcare professionals) to frontline emergency and intensive care. Established evidenced-based care pathways were modified to reduce length of stay where possible and to lower discharge thresholds to increase bed capacity within hospitals. Following the imposition of a national lockdown, the government advice to the public was to work from home and undertake essential travel only. Following this there was a reduction in the activity of the general population and therefore a reduction in the presentation of major and poly-trauma activity. However, the number of patients presenting with fragility hip fractures did not cease. The World Health Organisation stated in April 2020 that elderly patients are at highest risk from Covid-19 [2], and a recent study demonstrated an increased risk of mortality of 7.8% of people aged over 80 with an age-related gradient [3].

Studies have shown an increase in excess deaths by analysis of all-cause mortality, not just due COVID-19 (COVID) infection, during the first wave of the global COVID pandemic [4]. Non-COVID excess deaths are associated with increasing age, with the largest increases in non-COVID deaths being attributed to dementia and frailty. It is well known patients who present with fragility fractures are at risk of poorer outcomes [5,6][1, 2] and outcomes are care pathway dependent.

This study was designed to quantify the excess mortality related to the pandemic in this group following the modification of established care pathways in the light of the first wave. By maintaining long-established evidence-based care pathways for fragility fractures, even in the face of a future wave of the COVID-19 pandemic or future pandemics, excess death in this vulnerable population may be reduced.

Materials and Methods

A longitudinal cohort study design identified study and control groups from the same institution, a United Kingdom (UK) hospital group with a major trauma centre and 1700 inpatient beds. This was an analysis of prospectively collected data for both the control group and study group with data in both groups collected during patient admission as part of routine clinical care. The analysis of the control group was performed retrospectively since this was a pre-pandemic period which was used as a comparator. Inclusion criteria for both groups were all patients, of all ages, admitted with an ICD-10 code [7] of S72 (fracture of femur), M80 (osteoporosis with current pathological fracture), M96 (intraoperative and postprocedural complications and

For peer review only - http://bmjopen.bmj.com/site/about/guidelines.xhtml

disorders of musculoskeletal system, not elsewhere classified), W06 (fall from bed), W19 (unspecified fall) and Y79 (orthopaedic devices associated with adverse incidents). These ICD-10 codes were selected to capture patients who were likely presenting with fragility fractures, and were identified from data entered for the national Hospital Episode Statistics submission. Patients who present with fragility fractures have significant medical comorbidities, are elderly and frail. This group of patients are the ones at greatest risk of both Covid-19 and any alterations to established evidenced-based care pathways. Two time periods were selected to define groups: (i) the study group were admitted during the 1st wave of the UK COVID-19 pandemic, between 1 March 2020 to 1 June 2020, and discharged by 30 June 2020 (ii) the control group were admitted between 1 March 2019 to 1 June 2019, and discharged by 30 June 2019. The pandemic was declared by the World Health Organisation on the 11th of March 2019, but in the days before this there was an increase in the number of reported infections in the area served by our institution, so the study period was chosen from the 1st March 2019. Excluded were all patients that did not have one of the ICD-10 codes above. Mortality was established using NHS Digital data.

Demographic and injury data collected included patient age and sex, the diagnosis, COVID status (as identified in the medical records as "Coronavirus SARS-COV-2" positive patients or with a positive reverse transcriptase polymerase chain reaction; rPCR), number of comorbidities and whether or not the patient had a surgical procedure (as identified through the procedure codes of the submitted Hospital Episode Statistics data; HES). Covid testing was performed on admission for all patients and repeated every 3 days during inpatient stay, this was to facilitate and maintain safe distancing and isolation for covid contacts. Post discharge testing was not performed as these patients were either discharged home or to an institution. Hip fracture patients are not routinely followed up and those patients with other fragility fractures who are, were advised to not attend follow up if they developed symptoms or had a positive test.

For the subgroup of hip fracture patients, prospectively collected data was obtained from the institutional local hip fracture database. On this database, data for every adult patient admitted with a hip fracture is recorded using a modified version of the Standardised Audit of Hip Fractures in Europe (SAHFE) data collection form. Data analysed included mechanism of injury, hip fracture type, body mass index (BMI), abbreviated mental test score (AMTS,[8]), mobility status (Fracture Mobility Score [9]), Nottingham hip fracture score [10], haemoglobin concentration (Hb, as measured from the full blood count blood test) on admission and clinical frailty (as recorded on the Rockwood Clinical Frailty Scale; RCFS [11]). Data collected was confidential and managed in line with national data protection guidelines. Hospital outcome data were available for all patients and included type of surgery, mortality, requirement for invasive or non-invasive ventilation, length of stay and development of complications.

The data was extracted by a data analyst from our local database in July 2020 and ordered in Microsoft Excel (Redmond, WA, US) where numerical data comparison between groups was carried out using a student t-test

For peer review only - http://bmjopen.bmj.com/site/about/guidelines.xhtml

assuming a 2-tailed distribution with homoscedastic variance. For categorical data, where a 2x2 contingency table could be used, probability was calculated with the Fisher's exact test. For contingency tables greater than 2x2, the Chi-squared test was used. Statistical significance was set at $p<0.05$. Mortality was calculated from any death of any member of the cohort between 1st March and the 29th June in the respective year. Cox proportional hazards model were calculated using STATA 16.0 (StataCorp LLC, Texas, US) statistical software package, to compare survival between groups and to calculate the Hazard Ratio (HR) where the COVID 1st wave was the covariate. In addition, Student t-test (assuming a 2-tailed distribution with homoscedastic variance), Fisher exact test and Chi-squared test were used to calculate probability. The study was part of an approved local hospital audit (no. 20-333-C).

Patient and public involvement

Patients or the public were not involved in the design, or conduct, or reporting, or dissemination plans of our research.

Results

Demographic comparison between groups is shown in Table 1a. The percentage female and the number of hip fractures were equivalent between groups. Differences were noted with age and a lower percentage of patients receiving operative treatment during the COVID-19.

Hip fractures were the most common diagnoses making up approximately 20% in each of the two comparative groups. These were selected for a more granular subgroup analysis. Table 1b shows the variables used to assess selection bias between the hip fracture subgroups. No differences were seen between subgroups for the variables selected other than prevalence of COVID-19 infection.

Outcomes are recorded in Table 2, showing that mortality was significantly higher in the cohort presenting with a fragility fracture during the COVID-19 pandemic period. This was seen both in the COVID-19 period group as a whole and in the hip fracture sub-group analysis. It was also noted that the length of stay was significantly shorter for the COVID-19 period group and the COVID-19 period hip fracture subgroup.

Mortality of fragility fracture patients without COVID-19 was significantly higher among pandemic period admissions (14.7%) than in the pre-pandemic cohort (10.2%) after adjusting for age and sex (hazards ratio (HR)=1.86; 95%CI 1.41-2.45; $p<0.0001$) (Figures 1 and 2).

Table 3a shows variables that may have influenced outcomes. The cohort of COVID-19 positive patients was significantly older than the COVID-19 negative patients in the same time period. In relation to excess deaths in non-COVID patients, Table 3a also shows that the COVID-19 negative patients had a significantly shorter length of stay, lower frequency of operative treatment and less than expected ventilatory support than controls. There were no differences seen for sex or age between groups.

For peer review only - http://bmjopen.bmj.com/site/about/guidelines.xhtml

An analysis of hip fracture patients revealed a mortality of 8.4% among 190 admissions in the pre-pandemic set, and of 15.48% among 168 pandemic admissions with no COVID diagnosis, resulting in a HR=2.08 95%CI 1.11-3.90 p=0.021. After further adjustment for clinical frailty scores this became HR=2.15 95%CI 1.15-4.04 p=0.0162.

Discussion

In this study we report significantly higher mortality among fragility fracture patients with no COVID-19 related diagnosis admitted during the COVID-19 pandemic period compared to patients admitted with the same ICD-10 codes to the same hospital in the same time period of the year 2019. We also report significantly shorter hospital length of stay during the months of the pandemic compared to the same period a year earlier. These results hold true after adjustment for potential confounders such as age and sex. A sub-analysis on femoral neck fractures where frailty indices and other clinical assessments were readily available showed a similar pattern whilst there was no difference in frailty between the pandemic and pre-pandemic cohorts.

Limitations of this study include potential variables that have not been controlled that may have influenced the effects that have been seen. Knowledge of the fragility fracture patients that contracted COVID-19 in the community, who subsequently died within the period of assessment are not known and may account for a proportion of the excess mortality. In addition, it is known that during the first-wave period of investigation in this study, the institutional compliance with the best practice tariff was reduced. There is the potential that the redeployment of staff during the COVID-19 first wave, particularly the orthogeriatric support and physiotherapists, for fragility fracture patients, may have had a negative influence on outcomes. However, specific data about the effect of redeployment as a variable is not available for this study.

A recent report from the Office for National Statistics found that England had the highest overall relative excess mortality out of all the European countries compared during the first 6 months of 2020 [12]. While none of the four UK nations had a peak mortality level as high as Spain or the worst-hit local areas of Spain and Italy, excess mortality was geographically widespread throughout the UK during the pandemic, whereas it was more geographically localised in most countries of Western Europe. Our data add to this picture by showing that excess mortality involves patients without COVID-19 and areas of England with rates of infection below the national average. Achievement of best practice hip fracture tariff [13] at our institution during the pandemic fell from an average of 42% (Mar-Jun 2019) to 38% (Mar-Jun 2020) with a particular failure to achieve the delirium assessment, which fell from an average of 96% to 62% for the respective periods. In addition, there was a significant decrease in the number of hip fracture patients who were not delirious after surgery, from 81% to 63% respectively. This indicates breakdown of standard care pathways due to extrinsic pressures and provides a likely explanation for the excess mortality. As there are no agreed pathways or metrics for other fractures it is impossible to prove, although probable that the same factors apply.

Hip fractures occurred at the same frequency during the COVID-19 period and the national lockdown, as before it, with no differences identified for age, sex, BMI, AMTS, mobility, NHFS, anaemia, fracture type or frailty

For peer review only

between groups. Similar incidence pre- and post- COVID with similar demographics allows a valid comparison to examine excess deaths.

Analysis of the COVID-19 negative subset of those presenting during the COVID-19 period compared to the control period does identify associations that may be relevant. Whilst there were no differences identified between these groups for age, the development of ARDS or sepsis of the requirement for ventilatory support post procedure, significant difference was noted in the COVID-19 period group (COVID-19 negative subset) for a shorter length of stay and reduced number of procedures. Both differences suggest that the standard care pathways were altered for fragility fractures, which is further supported by the observed fall in BPT achievement. A reduced length of stay is often considered a positive outcome. However, if the threshold for a safe discharge is altered, for instance in the face of a great need for more inpatient beds to manage a pandemic, there may be unintended consequences affecting mortality. A significant proportion of patients with fragility fractures require input from health and social care in the form of rehab services and residential homes. During the pandemic period it was unknown regarding the virulence of spread of Covid-19 transmission within care homes and limiting testing in this group of people was available, this may have also influenced our mortality findings.

For those presenting with hip fractures during the COVID-19 period, who did not test positive for COVID-19, that no differences were seen for age, sex, BMI, treatment type (including non-operative treatment) and complications including: venous thromboembolism, urinary tract infection, stroke, myocardial infarction, failure of fixation or dislocation, transfusion, acute renal failure, pulmonary embolus or clostridium difficile when compared to the control group suggests that understanding the reasons for the excess mortality is complex. Despite this, reduction in length of stay seems to be a significant factor in the hip fracture subgroup analysis, where changes to operative treatment frequency (as for the fragility fracture group as a whole) is not. It has been demonstrated that a COVID-19 infection independently causes an increased mortality compared to those presenting with hip fractures that do not contract COVID-19 [14-16].

During the COVID-19 period, only 12 patients tested positive for COVID-19. When compared to those presenting during the same time period, they were older and had a longer length of stay. Age, as a risk factor for developing COVID-19, is well established [17].

During the Covid-19 pandemic period services were subjected to changes in both personnel and patient pathways. Although one may assume that there would have been a reduction in staff due to an increase in sickness and testing positive for Covid-19, the measures put into place did not impact the staffing levels and actually improved the staffing levels. Albeit at the expense of the following; trainee doctors did not rotate to other specialties or units to maintain continuity of care, clinicians out of programme for research / experience and research nurses were brought back into clinical practice, doctors were placed onto megarotas and staff normally working in the elective setting were moved to the acute setting to improve patient flow. The change in the established evidenced based guidelines for patient care pathways were altered to help improve patient

For peer review only - http://bmjopen.bmj.com/site/about/guidelines.xhtml

flow within the hospital, facilitate discharge and improve capacity for the at time unknown effect of the pandemic. These were approved both regionally and nationally by subspecialty working groups. [18]

These data suggest that excess deaths are likely due to changing established, evidence-based care-pathways, particularly regarding thresholds for safe discharge and length of stay. Excess mortality seen in this vulnerable fragility fracture population is likely to be minimised by maintaining standards of care during further waves of the COVID-19 or future pandemics.

Figure 1. Reduced survival in non-COVID-19 fragility fractures. Kaplan-Meier survival plot. Red COVID-19 pandemic (COVID-19 negative) fragility fracture patients. Blue pre-COVID-19 pandemic fragility fracture patients.

Figure 2. Reduced survival in non-COVID-19 fragility hip fractures. Kaplan-Meier survival plot. Red COVID-19 pandemic (COVID-19 negative) fragility hip fracture patients. Blue pre-COVID-19 pandemic fragility hip fracture patients.

For peer review only

References

1. ONS Deaths involving COVID-19, England and Wales - Office for National Statistics. https://www.ons.gov.uk/peoplepopulationandcommunity/birthsdeathsandmarriages/deaths/bulletins/deathsinvolvingcovid19englandandwales/deathsoccurringinjune2020 (accessed 27/07/2020).

2. Kluge, H. Statement – Older people are at highest risk from COVID-19, but all must act to prevent community spread. https://www.euro.who.int/en/health-topics/health-emergencies/coronavirus-covid-19/statements/statement-older-people-are-at-highest-risk-from-covid-19,-but-all-must-act-to-prevent-community-spread. (accessed 27/07/2020)

3. Verity, R.; Okell, L. C.; Dorigatti, I.; Winskill, P.; Whittaker, C.; Imai, N.; Cuomo-Dannenburg, G.; Thompson, H.; Walker, P. G. T.; Fu, H.; Dighe, A.; Griffin, J. T.; Baguelin, M.; Bhatia, S.; Boonyasiri, A.; Cori, A.; Cucunubá, Z.; FitzJohn, R.; Gaythorpe, K.; Green, W.; Hamlet, A.; Hinsley, W.; Laydon, D.; Nedjati-Gilani, G.; Riley, S.; van Elsland, S.; Volz, E.; Wang, H.; Wang, Y.; Xi, X.; Donnelly, C. A.; Ghani, A. C.; Ferguson, N. M., (2020) Estimates of the severity of coronavirus disease 2019: a model-based analysis. *Lancet Infect Dis*, ***20*** (6), 669-677.

4. Piccininni, M.; Rohmann, J. L.; Foresti, L.; Lurani, C.; Kurth, T., (2020) Use of all cause mortality to quantify the consequences of covid-19 in Nembro, Lombardy: descriptive study. *Bmj*, ***369***, m1835.

5. Buss, L.; McKeever, T. M.; Nightingale, J.; Akyea, R.; Ollivere, B.; Moppett, I. K.; Bolton, C. E., (2018) Hip fracture outcomes in patients with chronic obstructive pulmonary disease. *Br J Anaesth*, ***121*** (6), 1377-1379.

6. Ollivere, B.; Rollins, K.; Brankin, R.; Wood, M.; Brammar, T. J.; Wimhurst, J., (2012) Optimising fast track care for proximal femoral fracture patients using modified early warning score. *Ann R Coll Surg Engl*, ***94*** (4), 267-71.

7. World Health Organization: (1992) *The ICD-10 classification of mental and behavioural disorders : clinical descriptions and diagnostic guidelines*.

8. Hodkinson, H., (1972) Evaluation of a mental test score for assessment of mental impairment in the elderly. *Age and ageing*, ***1*** (4), 233-238.

9. Voeten, S. C.; Nijmeijer, W. S.; Vermeer, M.; Schipper, I. B.; Hegeman, J. H.; group, D. T. s., (2020) Validation of the Fracture Mobility Score against the Parker Mobility Score in hip fracture patients. *Injury*, ***51*** (2), 395-399.

10. Maxwell, M. J.; Moran, C. G.; Moppett, I. K., (2008) Development and validation of a preoperative scoring system to predict 30 day mortality in patients undergoing hip fracture surgery. *Br J Anaesth*, ***101*** (4), 511-7.

11. Rockwood, K.; Song, X.; MacKnight, C.; Bergman, H.; Hogan, D. B.; McDowell, I.; Mitnitski, A., (2005) A global clinical measure of fitness and frailty in elderly people. *Cmaj*, ***173*** (5), 489-95.

12. ONS, O. f. N. S. Comparisons of all-cause mortality between European countries and regions: January to June 2020 https://www.ons.gov.uk/peoplepopulationandcommunity/birthsdeathsandmarriages/deaths/articles/comparisonsofallcausemortalitybetweeneuropeancountriesandregions/januarytojune2020 (accessed 25/08/2020).

13. Royal-College-of-Physicians National Hip Fracture Database - Charts & Reports. https://www.nhfd.co.uk/20/NHFDCharts.nsf. (accessed 25/08/2020)

For peer review only

14. Kayani, B.; Onochie, E.; Patil, V.; Begum, F.; Cuthbert, R.; Ferguson, D.; Bhamra, J. S.; Sharma, A.; Bates, P.; Haddad, F. S., (2020) The effects of COVID-19 on perioperative morbidity and mortality in patients with hip fractures. *Bone Joint J*, *102-B* (9), 1136-1145.

15. Arafa, M.; Nesar, S.; Abu-Jabeh, H.; Jayme, M. O. R.; Kalairajah, Y., (2020) COVID-19 pandemic and hip fractures: impact and lessons learned. *Bone Jt Open*, *1* (9), 530-540.

16. Clement, N. D.; Ng, N.; Simpson, C. J.; Patton, R. F. L.; Hall, A. J.; Simpson, A. H. R. W.; Duckworth, A. D., (2020) The prevalence, mortality, and associated risk factors for developing COVID-19 in hip fracture patients: a systematic review and meta-analysis. *Bone Joint Res*, *9* (12), 873-883.

17. Li, X.; Xu, S.; Yu, M.; Wang, K.; Tao, Y.; Zhou, Y.; Shi, J.; Zhou, M.; Wu, B.; Yang, Z.; Zhang, C.; Yue, J.; Zhang, Z.; Renz, H.; Liu, X.; Xie, J.; Xie, M.; Zhao, J., (2020) Risk factors for severity and mortality in adult COVID-19 inpatients in Wuhan. *J Allergy Clin Immunol*, *146* (1), 110-118.

18. BOAST - Management of patients with urgent orthopaedic conditions and trauma during the coronovirus pandemic. https://www.boa.ac.uk/static/782e0b20-f9ce-4fc9-819f943740161405/201ebd61-5828-4c81-b45a8b80ac47fd50/COVID-19-BOASTs-Combined-v3FINAL.pdf (accessed 16th February 2022)

For peer review only

a.			
**Factor**	**COVID-19 period group (Admitted between 01/03/2020 - 01/06/2020) (n= 832)**	**Control period group (Admitted between 01/03/2019 - 01/06/2019) (n= 1014)**	**p value**
Mean age, years (± SD)	74.8 (± 20.0)	72.8 (± 21.4)	0.045[1]
Female, n (%)	492/832 (59.1%)	629/1014 (62.0%)	0.213[2]
rPCR COVID-19 positive, n (%)	104/832 (12.5%)	0/1014 (0%)	<0.00001[2]
Undergoing operative procedure, n (%)	70/832 (8.4%)	765/1014 (75.4%)	<0.00001[2]
Hip fracture, n (%)	180/832 (21.6%)	190/1014 (18.7%)	0.129[2]
**b.**			
**Factor**	**COVID-19 period hip fracture subgroup (total: n= 180)**	**Control period hip fracture subgroup (total: n= 190)**	**p value**
Mean age, years (± SD)	83.2 (± 8.8)	81.9 (± 9.2)	0.183[1]
Female, n (%)	132/180 (73.3%)	139/190 (73.2%)	1.000[2]
rPCR Covid positive, n (%)	12/180 (6.7%)	0/190 (0%)	<0.0001[2]
Mean BMI, Kg/m^2 (± SD); n	24.2 (± 5.2); 159	23.2 (± 4.4); 169	0.070[1]
Mean AMTS, score (± SD); n	6.9 (± 3.5); 179	6.7 (± 3.9); 189	0.538[1]
Mobility status, n (available; %)<br>• Freely mobile, no aids<br>• Outside with aids<br>• Unable to mobilise outside	n=175<br>61/175 (34.9%)<br>107/175 (61.1%)<br>7/175 (4.0%)	n=187<br>75/187 (40.1%)<br>96/187 (51.3%)<br>16/187 (8.6%)	0.076[3]
Mean Nottingham Hip fracture score (± SD); n	0.082 (±0.063); 172	0.081 (± 0.054); 181	0.838[1]
Anaemia on admission, Hb <12 g/L, n (%)	36/175 (20.6%)	45/182 (24.7%)	0.378[2]
Hip fracture type, n (%)<br>• Undisplaced intracapsular<br>• Displaced intracapsular<br>• Intertrochanteric<br>• Reverse oblique or subtrochanteric	 14/180 (7.8%)<br>76/180 (42.2%)<br>77/180 (42.8%)<br>13/180 (7.2%)	 16/190 (8.4%)<br>83/190 (43.7%)<br>79/190 (41.6%)<br>12/190 (6.3%)	 0.851[2]<br>0.398[2]<br>0.834[2]<br>0.837[2]

For peer review only - http://bmjopen.bmj.com/site/about/guidelines.xhtml

RCFS, mean category from 1-9 (± SD); n	4.8 (± 1.6); 155	4.6 (± 1.8); 175	0.421[1]

Table 1 a. Descriptive characteristics of all fragility fracture patients presenting during the two study periods. b. Hip fracture subgroup analysis for the two study periods rPCR (reverse transcriptase polymer chain reaction), BMI (body mass index), AMTS (abbreviated mental test score), Hb (whole blood haemoglobin), RCFS (Rockwood Clinical Frailty Scale); [1]Student t-test assuming a 2-tailed distribution with homoscedastic variance. [2]Fisher exact test. [3]Chi-squared statistic calculated on a 2x3 contingency table.

**a. Outcomes**	**COVID-19 period group (n= 832)**	**Control group (n= 1014)**	**Hazard Ratio (HR) and p value**
Mortality, n (%)	150/832 (18.0%)	104/1014 (10.2%)	HR 1.86; 95%CI 1.41-2.45; p<0.0001
Length of stay, mean days (± SD); n	8.8 (± 8.9), 832	12.1 (14.3), 1014	p<0.001[1]
**b. Outcomes**	**COVID-19 period hip fracture sub-group (n=180)**	**Control hip fracture subgroup (n=190)**	**Hazard Ratio (HR) and p value**
Mortality, n (%)	30/180 (15.5%)	16/190 (8.4%)	HR=2.08; 95%CI 1.11-3.90 p=0.021
Mortality adjusted for clinical frailty, n (%)	30/180 (15.5%)	16/190 (8.4%)	HR=2.15; 95%CI 1.15-4.04 p=0.0162
Length of stay, mean days, (± SD); n	11.1 (± 5.5); 180	15.3 (± 7.4); 190	p<0.001[1]

Table 2. Outcomes for each group. a. Outcomes for the full cohort. b. Outcomes for the hip fracture subgroup. SD (Standard Deviation), HR (Hazard Ratio), n (number). [1]Kruskal-Wallis test.

For peer review only

a.	COVID period group<br><br>COVID-19 positive<br><br>(total: n= 104)	COVID period group<br><br>COVID-19 negative<br><br>(total: n=728)	Control period group<br><br>(total: n= 1014)	p value
Age on admission; mean years (±SD); n	81.0 (±11.9)	73.9 (±20.7)		0.001[1]
		73.9 (±20.7)	72.8 (± 21.4)	0.301[1]
Male sex; n (%)	47/104 (45.2%)	292/728 (40.1%)		0.338[2]
		292/728 (40.1%)	385/1014 (38.0%)	0.370[2]
Length of stay; mean days (± SD); n	14.8 (±10.6)	8.0 (±8.3)		<0.001[3]
		8.0 (±8.3)	12.1 (±14.3)	<0.001[3]
Operative treatment; n (%)	12/104 (11.5%)	58/728 (8.0%)		0.255[2]
		58/728 (8.0%)	765/1014 (75.4%)	<0.00001[2]
ARDS or sepsis, n (%)	5/104 (4.8%)	26/728 (3.6%)		0.576[2]
		26/728 (3.6%)	26/1014 (2.6%)	0.254[2]
Ventilation post procedure, n (%)	6/104 (5.8%)	24/728 (3.3%)		0.253[2]
		24/728 (3.3%)	54/1014 (5.3%)	0.046[2]

For peer review only

b.	COVID period hip fracture subgroup<br><br>COVID-19 positive<br><br>(Total: n= 12)	COVID period hip fracture subgroup<br><br>COVID-19 negative<br><br>(Total: n= 168)	Control hip fracture subgroup<br><br>(Total: n= 190)	p value
Age on admission; mean years (±SD); n	82.5 (±6.3)	83.1 (±9.0)		0.818[1]
		83.1 (±9.0)	81.9 (± 9.2)	0.181[2]
Male sex; n (%)	4/12	44/168 (26.1%)		0.736[2]
		44/168 (26.1%)	51/190 (26.8)	0.905[2]
Length of stay; mean days (± SD); n	17.3 (±7.2)	10.7 (±5.15)		<0.001[3]
		10.7 (±5.15)	15.3 (± 7.4)	<0.001[3]
Hip fracture treatment				
• Arthroplasty	8/12	68/165* (41.2%)	81/190 (42.6%)	0.227[4]
• Fixation	4/12	90/165* (54.6%)	103/190 (54.2%)	0.355[4]
• Non-operative	0/12	7/165* (4.2%)	6/190 (3.2%)	0.587[4]
Pulmonary embolus or DVT	0/12	0/168	2/190 (1.1%)	
Pneumonia	3/12	14/168 (8.3%)	13/190 (6.8%)	0.081[4]
UTI	3/12	7/168 (4.2%)		0.021[2]
		7/168 (4.2%)	10/190 (5.3%)	0.804[2]

For peer review only

CVA	0/12	0/168	0/190	
MI or ACS	0/12	2/168 (1.2%)	0/190	
Dislocation or failure of fixation	0/12	0/168	1/190 (0.5%)	
Blood transfusion	2/12	32/168 (19.1%)	40/190 (21.1%)	0.856[4]
Renal failure	2/12	18/168 (10.7%)	22/190 (11.6%)	0.813[4]
Pressure ulcer	0/12	1/168 (0.6%)	1/190 (0.5%)	
Clostridium difficile	0/12	0/168	0/190	
BMI	24.7 (±3.5); 9	24.27 (±5.1);150		0.768[1]
		24.27 (±5.1);150	23.2 (± 4.4); 169	0.072[2]

Table 3. Complications, patient and treatment factors by group. [1]2-tailed distribution with homoscedastic variance Student's t-test. [2]Fisher exact test. [3]Kruskal-Wallis test. [4] Chi-squared statistic calculated on a 2x3 contingency table. * Data was only available for 165 patients.

For peer review only

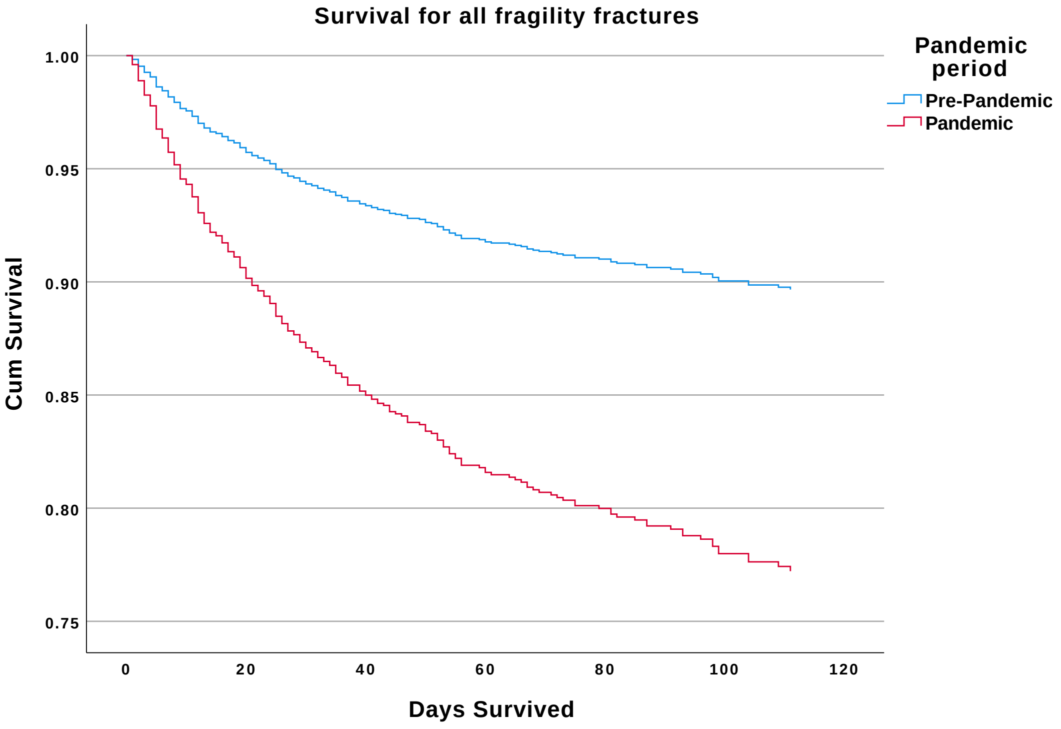

For peer review only - http://bmjopen.bmj.com/site/about/guidelines.xhtml

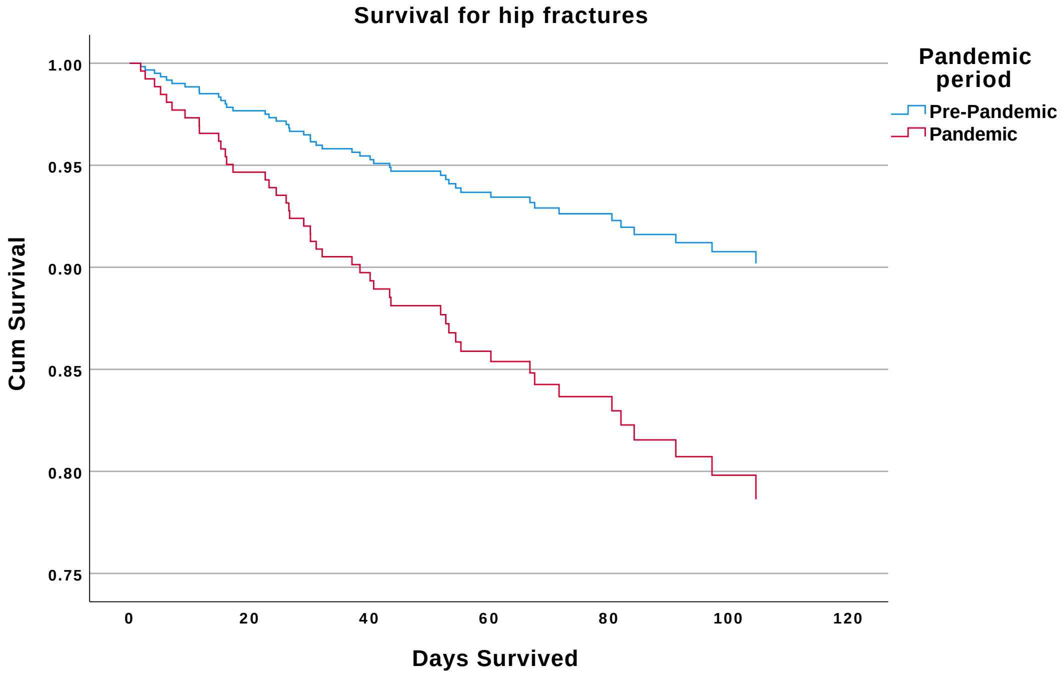

For peer review only - http://bmjopen.bmj.com/site/about/guidelines.xhtml

STROBE Statement—Checklist of items that should be included in reports of ***cohort studies***

	Item No	Recommendation	Page No
**Title and abstract**	1	(*a*) Indicate the study's design with a commonly used term in the title or the abstract	1
		(*b*) Provide in the abstract an informative and balanced summary of what was done and what was found	3
**Introduction**			
Background/rationale	2	Explain the scientific background and rationale for the investigation being reported	6
Objectives	3	State specific objectives, including any prespecified hypotheses	7
**Methods**			
Study design	4	Present key elements of study design early in the paper	7
Setting	5	Describe the setting, locations, and relevant dates, including periods of recruitment, exposure, follow-up, and data collection	7
Participants	6	(*a*) Give the eligibility criteria, and the sources and methods of selection of participants. Describe methods of follow-up	7-8
		(*b*) For matched studies, give matching criteria and number of exposed and unexposed	
Variables	7	Clearly define all outcomes, exposures, predictors, potential confounders, and effect modifiers. Give diagnostic criteria, if applicable	8
Data sources/ measurement	8*	For each variable of interest, give sources of data and details of methods of assessment (measurement). Describe comparability of assessment methods if there is more than one group	7-8
Bias	9	Describe any efforts to address potential sources of bias	9
Study size	10	Explain how the study size was arrived at	7
Quantitative variables	11	Explain how quantitative variables were handled in the analyses. If applicable, describe which groupings were chosen and why	8
Statistical methods	12	(*a*) Describe all statistical methods, including those used to control for confounding	8-9
		(*b*) Describe any methods used to examine subgroups and interactions	
		(*c*) Explain how missing data were addressed	
		(*d*) If applicable, explain how loss to follow-up was addressed	
		(*e*) Describe any sensitivity analyses	
**Results**			
Participants	13*	(a) Report numbers of individuals at each stage of study—eg numbers potentially eligible, examined for eligibility, confirmed eligible, included in the study, completing follow-up, and analysed	9, 16-21
		(b) Give reasons for non-participation at each stage	
		(c) Consider use of a flow diagram	
Descriptive data	14*	(a) Give characteristics of study participants (eg demographic, clinical, social) and information on exposures and potential confounders	9, 16-21
		(b) Indicate number of participants with missing data for each variable of interest	
		(c) Summarise follow-up time (eg, average and total amount)	
Outcome data	15*	Report numbers of outcome events or summary measures over time	9, 16-21

For peer review only - http://bmjopen.bmj.com/site/about/guidelines.xhtml

For peer review only

For peer review only - http://bmjopen.bmj.com/site/about/guidelines.xhtml

Main results	16	(*a*) Give unadjusted estimates and, if applicable, confounder-adjusted estimates and their precision (eg, 95% confidence interval). Make clear which confounders were adjusted for and why they were included<br>(*b*) Report category boundaries when continuous variables were categorized<br>(*c*) If relevant, consider translating estimates of relative risk into absolute risk for a meaningful time period	9,15-20
Other analyses	17	Report other analyses done—eg analyses of subgroups and interactions, and sensitivity analyses	9, 15-20
**Discussion**			
Key results	18	Summarise key results with reference to study objectives	10-12
Limitations	19	Discuss limitations of the study, taking into account sources of potential bias or imprecision. Discuss both direction and magnitude of any potential bias	10
Interpretation	20	Give a cautious overall interpretation of results considering objectives, limitations, multiplicity of analyses, results from similar studies, and other relevant evidence	10-12
Generalisability	21	Discuss the generalisability (external validity) of the study results	11-12
**Other information**			
Funding	22	Give the source of funding and the role of the funders for the present study and, if applicable, for the original study on which the present article is based	2

*Give information separately for exposed and unexposed groups.

Note: An Explanation and Elaboration article discusses each checklist item and gives methodological background and published examples of transparent reporting. The STROBE checklist is best used in conjunction with this article (freely available on the Web sites of PLoS Medicine at http://www.plosmedicine.org/, Annals of Internal Medicine at http://www.annals.org/, and Epidemiology at http://www.epidem.com/). Information on the STROBE Initiative is available at http://www.strobe-statement.org.

For peer review only

For peer review only - http://bmjopen.bmj.com/site/about/guidelines.xhtml
